# Corneal Epithelial Wound Healing Promoted by Verbascoside-Based Liposomal Eyedrops

**DOI:** 10.1155/2014/471642

**Published:** 2014-08-06

**Authors:** Luigi Ambrosone, Germano Guerra, Mariapia Cinelli, Mariaelena Filippelli, Monica Mosca, Francesco Vizzarri, Dario Giorgio, Ciro Costagliola

**Affiliations:** ^1^Department of Bioscience and Territory (DIBT), University of Molise, Contrada Lappone, Pesche, 86090 Isernia, Italy; ^2^Department of Medicine and Health Sciences, University of Molise, Via F. De Sanctis, 86100 Campobasso, Italy; ^3^Department of Public Health, University of Naples Federico II, Via S. Pansini 5, 80131 Naples, Italy; ^4^Department of Agriculture, Environment and Food (AAA), University of Molise, Via F. De Sanctis, 86100 Campobasso, Italy

## Abstract

Different liposomal formulations were prepared to identify those capable of forming eyedrops for corneal diseases. Liposomes with neutral or slightly positive surface charge interact very well with the cornea. Then these formulations were loaded with verbascoside to heal a burn of corneal epithelium induced by alkali. The cornea surface affected involved in wound was monitored as a function of time. Experimental results were modeled by balance equation between the rate of healing, due to the flow of phenylpropanoid, and growth of the wound. The results indicate a latency time of only three hours and furthermore the corneal epithelium heals in 48 hours. Thus, the topical administration of verbascoside appears to reduce the action time of cells, as verified by histochemical and immunofluorescence assays.

## 1. Introduction

Epidemiological, chemical, and clinical studies have provided various evidences that free-radical-induced oxidative damage of cellular membrane plays a causative role in aging and several degenerative diseases such as cancer, atherosclerosis, age related macular degeneration, and cataract formations [1–4].* In vitro* and* in vivo* evidence seem to indicate that antioxidants might have beneficial effects in protecting against these diseases. Thus it is not surprising that inhibition of free-radicals-induced oxidative damage, by means of antioxidant supplementations, has become a therapeutic strategy to reduce the risk of these diseases [5, 6]. The degree of oxidation and extent of the oxidative damage depend on the physical phase in which antioxidants are located and on the presence of interfaces [7–9]. Among others, phenyl-propanoid glycosides have been found to play important roles in protection against oxidative stress [10, 11]. Phenylpropanoid glycosides are water-soluble derivatives of natural polyphenols widely distributed in the plant kingdom. Verbascoside belongs to the phenylpropanoid glycoside group and is structurally characterized by caffeic acid and 4,5-hydroxyphenylethanol bound to a *β*-(D)-glucopyranoside, with a rhamnose in sequence (1–3) to the glucose molecule. Although there are many studies concerning the biological activity of verbascoside, its molecular mechanism and target are uncertain. Recently, we showed that a prolonged verbascoside-based diet improves both health status and the oxidative state of the different eye tissues in rabbits and hares [12, 13]. In these experiments verbascoside was administered in the form of tiny capsules of lipids for effectively protecting the antioxidant during its passage in the digestive tract [13]. It is well documented that verbascoside promotes skin repair and ameliorates skin inflammation [14]. Traditionally, ocular drug therapies have been administrated in the form of topical eyedrops. However, using this means of delivery, the drug may be quickly eliminated due to overflow and tear drainage [15]. Following the instillation of a normal 50 *μ*L eyedrop, approximately 20–30 *μ*L is immediately lost to overflow since the maximum volume that can be retained in the eye is 20–30 mL when blinking is prohibited or 10 *μ*L when blinking is permitted [15, 16]. In order to improve the therapeutic benefit and to confer to liposome a specificity for a certain cell or organ macromolecules such as antibodies, peptides and ligands of natural receptors are conjugated on liposome [17, 18]. The aim of this study has been to verify the efficacy of a verbascoside-based liposomal eyedrops in alkali corneal wound and to ascertain the corneal retention liposomal affinity toward liposomes by modulating their surface charge.

## 2. Material and Methods

### 2.1. Chemicals

Soy lecithin (phosphatidylcholine-enriched fraction, Epikuron 200), composed of phosphatidylcholine (min. 92%), lysophosphatidylcholine (max. 3%), other phospholipids (max. 2%), and fatty acid (approximately 1%), was kindly offered by Cargill, Inc. The average fatty acid composition of this lecithin says that the linoleic acid is the most abundant (≈60%) followed by palmitic and oleic acid. Phospholipids Epikuron 130 P composed of phosphatidylcholine (33%), phosphatidylethanolamine (15%), phosphatidylinositol (16%), and phosphatidic acid (6%) was kindly offered by Cargill. Cholesterol from lanolin was purchased from Fluka Analytical. Any other reagent used was of analytical grade of purity.

### 2.2. Preparation of Liposomal Encapsulated Verbascoside

Liposomes with different sizes and surface charges were used. They were prepared from lecithin and conventional rotary evaporation-sonication method [19]. Appropriate amounts of lecithin (40 mg) were dissolved in chloroform. The mixture was dried to a thin film under vacuum. The film was then hydrated with phosphate buffer (10 mM, pH 7.4) to make a 20 mL of lipid coarse dispersion. Cholesterol was added in a 4 : 1 lecithin-cholesterol molar ratio, PC/C liposomes. Sonication was carried out at 15°C and under N_2_ (water bath sonicator, P-Selecta Ultrasons, 60 Hz) on 3 mL aliquots of the coarse dispersion. Then, 300 *μ*L of this sonicated dispersion was diluted to 3 mL in phosphate buffer and sonicated for 10 min at 15°C in N_2_ to obtain liposomes with a size of about 200 nm. The final lecithin concentration of all the final dispersions is 0.2 mg/mL. This procedure of preparation by sonicator was found by monitoring the size and *z*-potential with sonication time through preliminary experiments. The method described above was used to prepare two types of liposomes useful for formulations of eyedrops. The first contains lecithin Epikuron 200 (EP200); the other one contains phospholipids Epikuron 130 P (EP130).

### 2.3. Size Measurement and *z*-Potential

Liposomes size was measured by a dynamic light scattering (DLS) particle size analyzer which has a measuring range from 0.6 nm to 6 *μ*m (Zetasizer Nano ZS90, Malvern Instruments Ltd., Worcestershire, UK). Dynamic light scattering, also known as photon correlation spectroscopy (PCS), measures Brownian motion in relation to the particles size: by illuminating the particles with a laser and analyzing the intensity fluctuations in the scattered light. The relationship between the size of a particle and its speed due to Brownian motion is defined by the Stokes-Einstein equation. The final particle diameter was calculated from a mean of at least three measurements. DLS measurements also provide the polydispersity index (PDI), which allows us to evaluate how the size of liposome population is distributed around a mean diameter [20]. The *z*-potential was measured using the Zetasizer Nano ZS90, which measures the distribution of the electrophoretic mobility of particles with a size range from 3 nm to 10 *μ*m using the laser Doppler velocity technique. Since the *z*-potential is related to the electrophoretic mobility of the particles, the analyzer calculates the *z*-potential from the measured velocity using the Smoluchowski approximation, valid in the case of aqueous solutions.

### 2.4. Encapsulation Efficacy

Encapsulation efficiency was determined as the percentage verbascoside encapsulated in liposome to the original amount of verbascoside added. To determine drug release efficiency of liposome, lipid vesicles were lysed using 100% Triton X-100. Briefly, 100 *μ*L of liposomal suspension was added to 100 *μ*L 100% Triton X-100 and vortexed for 5 min to ease lysis of the liposomal encapsulated verbascoside. Free verbascoside was separated from liposome by centrifugation. Concentrations of the verbascoside in the filtrate, total drug, and free drug were quantitatively analyzed using spectrophotometric peak at 328 nm. The encapsulation efficacy was calculated using the following formula:

(1) $$\begin{array}{c} \eta =\frac{T-F}{T}\cdot 100, \end{array}$$

where *η* is the efficiency of encapsulation, *T* the total verbascoside for encapsulation, and *F* free drug in the sample.

### 2.5. Alkali Burn Procedure

Adult hares of either sex weighing 3.0–3.5 kg were used in this study. All procedures were performed according to the ARVO Statement for the Use of Animals in Ophthalmic and Vision Research. Hares eyes were anesthetized topically with two drops of proparacaine hydrochloride (Alcon Laboratories, Ft. Worth, TX). One eye of each animal received a corneal burn by pipetting 0.5 mL of 2 N NaOH [21]. External examinations of each hares cornea were performed daily. Examinations of the alkali burns were performed every morning controlling the presence of corneal defects, ulceration, perforation, vascularization, or infection. Generally, corneal opacity is classified under a dissociation microscope as follows: 0, no opacity; 1, less than one-third of the corneal surface being clouded; 2, less than two-thirds of the corneal surface being clouded; 3, more than two-thirds of the corneal surface being clouded; and 4, almost all the corneal surface being clouded, and the opacity prevents visualization of the pupil margins. According to this classification, our system is of class 1. However such a classification is not necessary for kinetic analysis performed in this research so that it has not been used. For each animal just one eye was used for analysis. Animals were randomly assigned into two groups; one group of three hares received one treatment with liposomal eyedrop containing verbascoside daily, while the other group (three animals) received treatment with liposomal eyedrop without verbascoside as controls. Eyes of both groups were followed morphologically with taking photos of the corneas.

### 2.6. Histochemistry

Corneal fragments obtained from hares were fixed in buffered 10% formalin, embedded in paraffin, and sectioned. 5 *μ*m thick serial sections of corneal specimens were deparaffinized and treated for hematoxylin and eosin (H&E) routine staining (haematoxylin: Fluka, AG, Switzerland, Buchs SG-Eosin Y: alcohol and water soluble, Winlap, UK).

### 2.7. Apoptosis Assay

In corneal epithelium apoptotic cells were detected using a commercially available fluorescence kit (ApopTag Plus Fluorescein* in situ* Apoptosis Detection Kit, Chemicon International, Temecula, CA, USA) based on the TUNEL method, which detects and labels the free 3′-OH end of DNA strand breaks in apoptotic nuclei. According to the manufacturer's protocol, sections were fixed in 1% PFA solution after washing with PBS. Digoxigenin-labeled nucleotides in reaction buffer and terminal deoxynucleotidyl transferase enzyme (TdT) were applied to the sections for one hour at 37°C to catalyze the template-independent addition of nucleotide triphosphates to the 3′-OH ends of double-stranded or single-stranded DNA. After termination of the reaction, fluorescent-labeled antidigoxigenin antibodies were applied to visualize the nucleotides added to DNA free ends. Sections were counterstained with DAPI and visualized using fluorescence microscopy [22, 23]. Microscopic analysis was performed with a Leica DMLB microscope equipped with epifluorescence EL6000 system (Leica Microsystems, Solms, Germany). Images were captured with a CCD camera (DC 200, Leica Microsystems, Solms, Germany) and image analysis software Quantimet 520 (Leica Microsystems, Solms, Germany). The number of apoptotic nuclei that stained intensely green was expressed relative to total number of nuclei stained by DAPI.

### 2.8. Analysis of Data

Image analysis involves the conversion of features and objects in image data into quantitative information about these measured features and attributes. Digital images of corneas were acquired using a CCD camera (DC 200, Leica, Solms, Germany). The contours in the images appeared to be always well-distinguishable so that any filtering was not necessary; then we applied computational techniques to extract the corneal-wound area from the images. MATLAB tools have been used to measure the area of the regions affected by the alkali burn. The area of the corneas of untreated eyes was used to calibrate the method and determine the scaling factor.

## 3. Results

### 3.1. Size Measurement and *z*-Potential

Size, PDI, and *z*-potential were measured for liposomes EP200 and EP130, both before and after the encapsulation of verbascoside. Results are collected in Table 1.

As one can see, for both formulations liposomes size is reduced after the inclusion of verbascoside and this indicates that the phenylpropanoid is involved in the assembly process. PDI values in Table 1 suggest that the size distribution of liposomes is very narrow and this monodipersity does not vary for the inclusion of guest molecules. Liposomes EP130 exhibit a net negative charge whilst EP200 liposomes are positive and become practically neutral after the inclusion of verbascoside. For this reason, we decided to prepare eyedrops with Liposomes EP200. For these formulations a further filtering step was performed (Whatman filters) and the residual moisture was removed in laminar-flow-hood to avoid contamination.

### 3.2. Morphological Analysis

**Figure 1:**
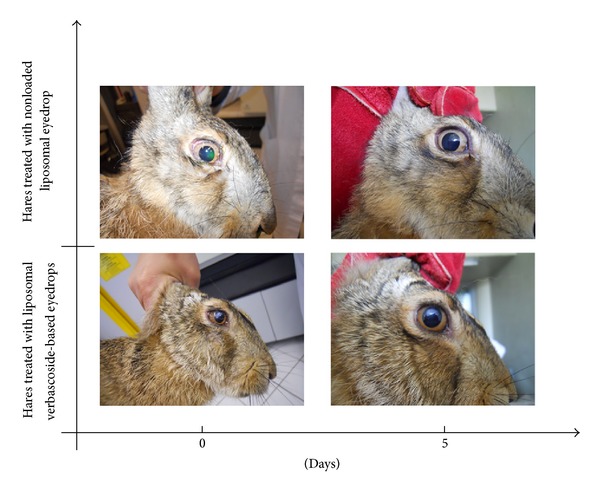
Morphological comparison between alkali burns in hares treated with verbascoside loaded liposomal eyedrops and hares treated only with liposomes, as a function of time.

Figure 1 shows the morphology of the corneal wound at time zero (i.e., just formed) and after four days, for both groups of hares.

**Figure 2:**
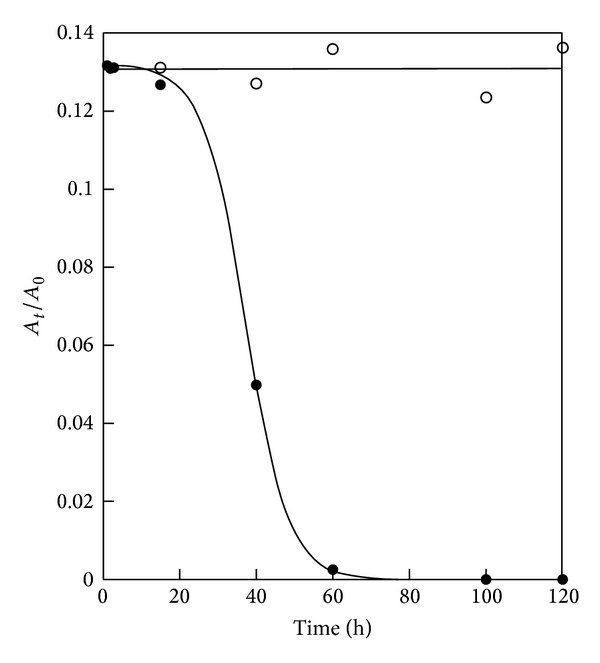
Fraction of cornea burned as a function of time for hares treated with verbascoside loaded liposomal eyedrops (•) and hares treated only with liposomes (○).The curve is the fit of (5) to the experimental data.

It can be seen that the wound is almost circular in shape and occupies 15% about of cornea surface and involves only epithelial layers. After four days animals treated with verbascoside-based eyedrops have a perfectly healthy cornea; on the contrary animals treated with only liposomes exhibit an unchanged wound both in shape and in size. Since the extension of a wound is directly proportional to the degree of inflammation, we decided to monitor the evolution of corneal burning with measuring wound surface, *A*, as a function of time. However, the measured surface is not planar and therefore one should introduce corrective terms to take into account the true shape. To avoid the introduction of unknown parameters, it is useful to define the ratio *A*/*A*_*c*_, being *A*_*c*_ the surface area of entire and healthy cornea. In other words we monitor the fraction of cornea surface involved in the ulceration process. Results are displayed in Figure 2, for both groups of hares. It is evident that the treatment with only liposomes has no effect and then *A*/*A*_*c*_ remains constant. For animal treated with verbascoside-based liposome the ratio remains constant only for the first 2-3 hours and then decreases to become zero.

### 3.3. Histochemistry

**Figure 3:**
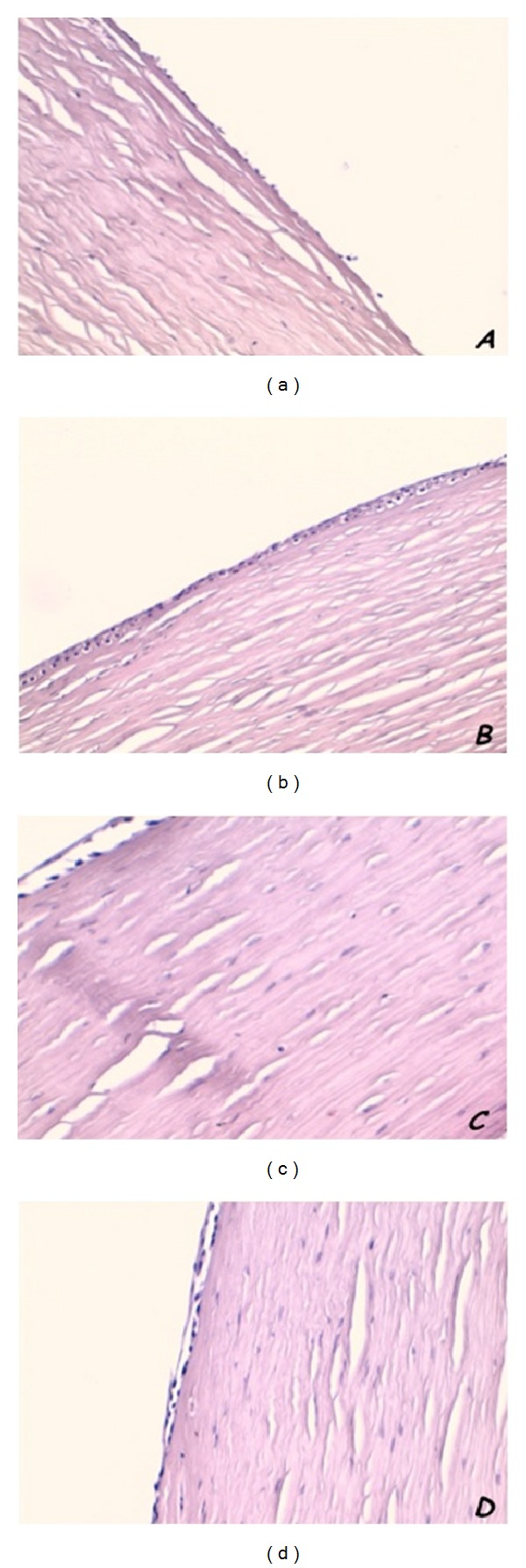
H&E staining in hares treated with verbascoside loaded liposomal eyedrops and hares treated only with liposomes. After alkali burn corneal epithelium appears thinner than normal and with superficial layer completely removed ((a)–(c)). Epithelial layer shows a restored normal thickness after treatment (b) but in untreated animals appears still thinner (d). Original magnification ×20.

The hematoxylin and eosin (H&E) combination is the most common staining technique used in histology. Hematoxylin has a deep blue-purple color and stains nucleic acids by a complex, incompletely understood reaction. Eosin is pink and stains proteins nonspecifically. In a typical tissue, nuclei are stained blue, whereas the cytoplasm and extracellular matrix have varying degrees of pink staining. H&E staining shows in corneal samples obtained from hare's eye injured by alkali burn a completely damaged epithelial layer. In Figures 3(a) and 3(c) superficial layer appears completely removed and polygonal cell of deep layer reduced in number and disorganized. After treatment with verbascoside-based eyedrop epithelial layer looked completely reorganized. Epithelial layer observed using H&E staining showed normal thickness and architecture (Figure 3(b)). In untreated hare's eye corneal epithelium looks still damaged with a superficial as well as deep layer being thinner and unorganized (Figure 3(d)).

### 3.4. Apoptosis Assay

**Figure 4:**
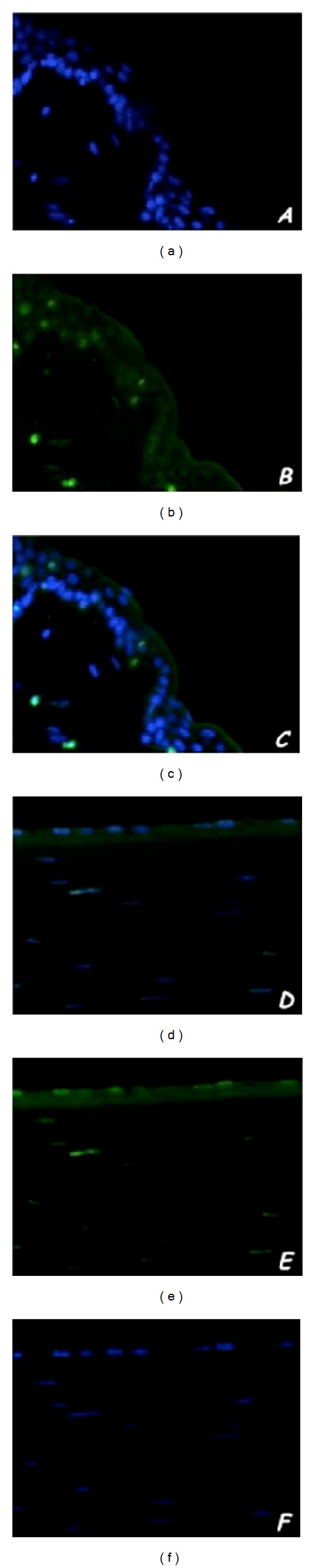
Representative images of nick end-incorporated nucleotides (for evaluation of apoptosis) immunofluorescent green staining in hares treated with verbascoside loaded liposomal eyedrops. Alkali burn induces in epithelial cell an increasing intensely green nuclear apoptotic staining (a) comparing normal nuclear staining with DAPI (b); merge image shows a plastic picture of this phenomenon (c). Verbascoside treatment shows a large reduction of intensely green apoptotic epithelial cells (d) with respect to nonapoptotic cells stained with DAPI (e); merge image represents clearly this condition (f). Original magnification ×20.

*In situ* ApopTag Plus Fluorescein* in situ* Apoptosis Detection Kit (Chemicon International, Temecula, CA, USA) based on terminal transferase dUTP nick end labeling was used to evaluate the apoptosis of corneal epithelial cells. The number of apoptotic nuclei that stained intensely green was expressed relative to total number of nuclei stained by DAPI. In corneal samples obtained from hare's eye injured by alkali burn a large number of intensely green stained cells were described (Figures 4(a)–4(c)). Treatment by verbascoside-based eyedrop induced a large reduction of apoptotic phenomenon in corneal epithelium. Figures 4(d)–4(f) show only few green stained cells. In untreated samples no changes in apoptosis assay were observed.

## 4. Discussion

Following trauma to the corneal epithelium, the restoration of epithelial cell layers is crucial to the maintenance of normal visual acuity. Experimental study on corneal epithelial wound closure suggests that the process includes two distinct phases, an initial (or* latent*) phase followed by a closure wound phase. The latent phase, which has been found to last between 5 and 6 hours in both rabbit and monkey, is characterized by wounded-triggered cellular reorganization processes, including desquamation, loss of columnar appearance of the basal layers of cells, and breakdown of hemidesmosomes at the basal membrane [15, 16, 24]. During this phase little or no wound closure is observed. At the onset of the closure phase, the leading edge of the transformed epithelium is composed of a single layer of cells. Epidermal growth factor (EGF), keratinocyte growth factor, vascular endothelial factor (VEGF), and platelet derived growth factor (PDGF) are some of the growth factors known to stimulate corneal wound healing. These factors have been shown to promote corneal epithelial cell migration and wound closure* in vivo*. Epidermal growth factor (EGF) is also used to treat alkali-burned corneas. However, EGF-induced corneal angiogenesis, which is currently untreatable, is a side effect of this therapy. It has been recently demonstrated that blockade of the intermediate-conductance (Ca^2+^) activated K^+^ channel inhibits the angiogenesis induced by epidermal growth factor in the treatment of corneal alkali burn [25]. Ca^2+^ plays a master role in the complex and multistep process of angiogenesis by regulating endothelial proliferation, migration, adhesion to the substrate, contractility, and organization into capillary-like structures in normal [25–29] and neoplastic conditions [30–34]. Results displayed in Figure 2 seem to confirm a two-stage mechanism of wound healing also for hares. Moreover, it should be noted that the latency exhibited in Figure 2 is due to not only the cellular reorganization, but also the accumulation of liposomes on the wound boundary. To interpret the results displayed in Figure 2, we assume that the rate of wound healing contributes gain and loss factors. Schematically, we write

(2) $$\begin{array}{c} \frac{dx}{dt}=\text{gain}-\text{loss}, \end{array}$$

where *x* = *A*/*A*_*c*_. The left side of (2) represents the rate of healing. The gain depends both on the migration of epithelial cells from periphery of the wound into the wound region and on the diffusion of liposomes which release verbascoside. Therefore, we can assume the gain to be a second-order process:

(3) $$\begin{array}{c} \text{gain}=k_{2}x^{2}, \end{array}$$

where *k*_2_ is a kinetic constant. The loss, however, being proportional to verbascoside molecules reacting, is a process of first order:

(4) $$\begin{array}{c} \text{loss}=\frac{x}{\tau }, \end{array}$$

where *τ* is the lifetime [35, 36] of the closure process.

Therefore (2) becomes

(5) $$\begin{array}{c} \frac{dx}{dt}=-\frac{x}{\tau }+k_{2}x^{2}. \end{array}$$

Which, solved with the initial condition *x*(0) = *x*_0_, provides

(6) $$\begin{array}{c} x\left(t\right)=\frac{x_{0}\left(1+B\right)}{1+Be^{-t/\tau }}, \end{array}$$

where

(7) $$\begin{array}{c} B=\frac{1}{\tau k_{2}x_{0}}-1. \end{array}$$

In computational terms, parameters *τ* and *B* were calculated by nonlinear fitting of (5) to experimental data while *x*_0_ was directly measured. The values of chi-square (*χ* ^2^ = 2.6 · 10^−5^) and correlation coefficient (*R* = 0.9995) indicate that the model fits very well the experimental data. By applying this procedure we get *τ* = (12.1 ± 0.7) h and *k*_2_ = 0.60 ± 0.05 h^−1^. As seen from (5), when wound surface is such that *x*_0_ ≪ 1/*τk*_2_, *x*(*t*) remains constant. Furthermore, for both groups of animals, the experimental curves start from the same values so that we deduce that phenylpropanoid molecules reduce the latency time by lowering the lifetime *τ*. Using these parameters one obtains that in 48 hours the wound heals. This result is remarkable when compared with the value of 40 hours obtained under continuous delivery of EGF [24]. Damage to the corneal epithelium can be caused by trauma, microbial insult, or chemical insult, during contact lens wear or by surgery such as photorefractive keratectomy or laser* in situ* keratomileusis. Moreover, degenerative corneal disease such keratoconus is characterized by a thinning of the central part of epithelium [37]. Most corneal epithelial wounds heal promptly. However, under certain clinical conditions, such as chemical injury, healing of the corneal epithelium is delayed, leaving the underlying stroma vulnerable to infection and ulceration. Alkali injuries are of particular concern and cause acute inflammation characterized by rapid infiltration of neutrophils into the cornea followed by chronic inflammation involving the migration and recruitment of inflammatory cells over extended periods, further damaging the corneal surface. Oxidative stress plays an important role in pathogenesis of several corneal diseases. Corneas are characterized by the disturbed lipid peroxidation and nitric oxide pathways. Malfunctioning of these pathways may lead to accumulation of their toxic by-products inducing several detrimental effects, along with apoptosis of the epithelial corneal cells. Reactive oxygen species (ROS) are the prime initiators of the angiogenic response after alkali injury of the cornea. Light microscopy histochemical analysis performed using H&E routine staining showed in corneal samples obtained from hare's eye injured by alkali burn an epithelial layer almost completely destroyed. Epithelial cells of superficial layer appeared completely removed. A large reduction in number and a loss of regular organization and connections of epithelial polygonal cell of deep layer were also observed. Nevertheless, in our samples alkali burn induced in epithelial cells an increasing intensely green nuclear apoptotic staining comparing normal nuclear staining with DAPI observed in nonapoptotic cells. Nanotechnology provides the opportunity to design and develop drug delivery systems able to target and treat several diseases, including those mediated by inflammation. Up to date, several delivery systems have been designed to deliver drugs to the retina. Drug delivery strategies may be classified into 3 groups: noninvasive techniques, implants, and colloidal carriers. Colloidal systems (liposomes, nanoparticles, etc.) can be easily administrated in a liquid form. Nanostructured nanolipids carriers are biocompatible, are easy to produce at large scale, and may be autoclaved or sterilized.

## 5. Conclusion

The positive influence of a prolonged diet supplemented with the powerful antioxidant verbascoside on the oxidative state in hares was recently demonstrated by our group. The research established that verbascoside supplementation is able to protect ocular tissue and fluids from naturally occurring oxidation and that its protective effect depends on the daily dose, being maximum up to 3 *μ*g/die. Feed administration of verbascoside exerts higher antioxidant capacity in retina, lenses, and optic nerve. In present research we utilized topic administration of verbascoside-based eyedrops. After treatment we performed H&E staining to demonstrate a complete reorganization of epithelial layer. Corneal epithelium showed normal thickness and restored architecture of all layer. Histochemical analysis of untreated hare's eye displayed a corneal epithelium which is still damaged with a superficial as well as deep layer being thinner and unorganized. In treated animals nick end-incorporated nucleotides immune-fluorescent green staining for evaluation of apoptosis showed a large reduction of intensely green apoptotic epithelial cells with respect to nonapoptotic cells stained with DAPI. The number of apoptotic corneal epithelial cells does not change in comparison to number of cells died by apoptosis induced by alkali burn in untreated animals. The results show that neutral liposomes interact well with the cornea and fail to deliver suitable amounts of verbascoside relatively quickly. A mathematical model based on the idea that the area burned by alkali is proportional both to the number of cells that arrives from the periphery and to the amount of verbascoside that, loaded in liposomes, is suggested. The model fits well experimental data and the curves obtained indicate that topical administration of verbascoside reduces significantly the first stage of the process of wound healing of the corneal epithelium.

## Figures and Tables

**Table 1:** Experimental results of different liposomal formulations.

	EP130			EP200		
	*z*_av_ (nm)	*z*-Potential (mV)	PDI	*z*_av_ (nm)	*z*-Potential (mV)	PDI
Liposomes	135	−25.0	0.117	107	−7.5	0.104

Liposomes containing verbascoside	113	−26.2	0.152	112	+4.8	0.095
